# Contributions of lunate cells and wax crystals to the surface anisotropy of *Nepenthes* slippery zone

**DOI:** 10.1098/rsos.180766

**Published:** 2018-09-05

**Authors:** Lixin Wang, Dashuai Tao, Shiyun Dong, Shanshan Li, Yu Tian

**Affiliations:** 1 School of Mechanical Engineering, Hebei University of Science and Technology, Shijiazhuang 050018, People's Republic of China; 2 State Key Laboratory of Tribology, Tsinghua University, Beijing 100084, People's Republic of China; 3 National Key Laboratory for Remanufacturing, Academy of Armored Forces Engineering, Beijing 100072, People's Republic of China

**Keywords:** surface anisotropy, *Nepenthes* slippery zone, lunate cell, wax crystals, friction data

## Abstract

*Nepenthes* slippery zone presents surface anisotropy depending on its specialized structures. Herein, via macro–micro–nano scaled experiments, we analysed the contributions of lunate cells and wax crystals to this anisotropy. Macroscopic climbing of insects showed large displacements (triple body length within 3 s) and high velocities (6.16–20.47 mm s^−1^) in the inverted-fixed (towards digestive zone) slippery zone, but failed to climb forward in the normal-fixed (towards peristome) one. Friction force of insect claws sliding across inverted-fixed lunate cells was about 2.4 times of that sliding across the normal-fixed ones, whereas showed unobvious differences (1.06–1.11 times) between the inverted- and normal-fixed wax crystals. Innovative results from atomic force microscope scanning and microstructure examination demonstrated the upper layer of wax crystals causes the cantilever tip to generate rather small differences in friction data (1.92–2.72%), and the beneath layer provides slightly higher differences (4.96–7.91%). The study confirms the anisotropic configuration of lunate cells produces most of the anisotropy, whereas both surface topography and structural features of the wax crystals generate a slight contribution. These results are helpful for understanding the surface anisotropy of *Nepenthes* slippery zone, and guide the design of bioinspired surface with anisotropic properties.

## 1. Introduction

*Nepenthes* plants have evolved pitchers growing from the tips of their leaves to capture and digest insects, which provide nutrients to survive in the infertile habitats [1–4]. Considering the significant differences in macromorphology, microstructure and their corresponding functions, the pitchers can be typically divided into four parts: a canopy-like lid, a collar-formed peristome, a slippery zone and a digestive zone [5,6]. The canopy-like lid prevents the inner pitcher from being contaminated [7], also acts as a catapult to make insects fall into the pitcher below [8]. The collar-formed peristome consists of radial ridges, which extend towards the pitcher inside [2,9,10]. These structures make liquid film generate a continuously unidirectional transport [11], having been considered as a bionic prototype to design functional surfaces for the unidirectional liquid transport [12–15], and to develop super-lubricant interfaces for the controllable motion of liquid droplets [16–20]. The slippery zone contains a great number of lunate cells and a layer of wax crystals [6,21]. Having the function of trapping prey [22], the structures have attracted studies to investigate their anti-attachment mechanism [23–26], and to develop bioinspired surfaces with anti-adhesive or hydrophobic properties [27–31]. The digestive zone has been well explored in digestion of prey, absorption and transport of the derived nutrients [32,33].

A large number of studies have focused on the surface anisotropy of *Nepenthes* slippery zone. To explore the potential role of surface anisotropy in restricting attachment ability of insects, authors initially postulated that the lunate cells depend on their crescent-shaped projections with asymmetrical profiles to allow grasping when being turned upside down [34]. However, the subsequent experiments could not present such attachment behaviours [35]. By behaviour observations, Gaume *et al*. [7] investigated the anisotropic effect of lunate cells on insect locomotion, and postulated that the lunate cells are probably responsible for the anisotropic frictional properties of slippery zone, as enhancing/restricting attachment capability in the downward/upward direction. Further behaviour observations confirmed that the convex lunate cells cannot provide any sites for claw anchorage in the upward orientation, whereas such structures may provide sites for claw anchorage to make flies *Calliphora vomitoria* climb across the slippery zone in the downward orientation [22]. However, these studies only presented the statistical information of insects which fail or succeed to climb on the slippery zone. Recently, Gorb & Gorb [36] studied the influence of surface anisotropy on insect attachment ability. Convincible results mainly derived from traction force measurements have demonstrated that the lunate cells depend on their particular morphology to make the slippery zone present observable anisotropic properties, causing the ladybird beetle *Coccinella septempunctata* to generate rather high traction forces in the downward direction. Zhang *et al*. [37] showed strong evidence to further understand the anisotropic structures of lunate cells. They found that just about 6% of the experimental ants were able to escape from the upward-placed slippery zone, whereas about 58% escaped when the slippery zone was inverted. Also, the friction forces generated by climbing crickets in the slippery zone along the downward direction are two to three times those along the upward direction. According to these results, they considered the lunate cells depend on their anisotropic structures to greatly affect insect attachment ability. These above studies have described the anisotropic behaviours of slippery zone, in terms of insect attachment behaviours, and assumed that the lunate cells were the causation. However, only statistical information was presented in climbing behaviours, and only peak values of the traction/friction force were shown in traction/friction force measurement. The procedure information, such as displacement, velocity and real-time force variation, are rather necessary to definitely characterize the anisotropic properties. Further, they have not presented evidence to include or exclude the contribution of wax crystals to the surface anisotropy yet.

In this present study, via macro–micro–nano experiments, including climbing behaviour observation, friction force measurement, atomic force microscope (AFM) scanning and scanning electron microscope (SEM) examination, we showed procedure information about climbing behaviours and friction forces, especially the innovative information about friction data provided by an AFM cantilever, to systematically analyse the contributions of lunate cells and wax crystals to the surface anisotropy of slippery zone. Based on the results, the anisotropy mechanism of *Nepenthes* slippery zone can be well revealed, and the bioinspired surface with fine anisotropic properties can be developed.

## 2. Material and methods

### 2.1. *Nepenthe*s pitchers and insects

**Figure 1.**
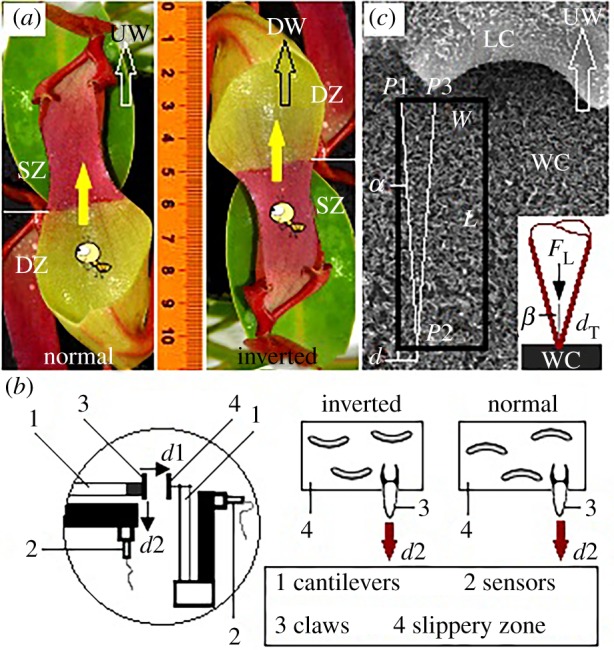
Experimental descriptions. (*a*) Climbing behaviours observation. Insects climbed forward on the normal- and inverted-fixed pitchers, equalling to along upward (UW, towards peristome) and downward (DW, towards digestive zone) directions, and the starting region was the digestive zone (DZ) and the slippery zone (SZ), respectively. (*b*) Schematic of force measurement part in the friction measurement apparatus (electronic supplementary material, figure S1). The slippery zone was fixed in its normal (upward) and inverted (downward) direction, respectively. *d*1, direction of loading a normal force; *d*2, direction of sliding insect claws to generate the friction force. (*c*) Scanning trajectories of the AFM cantilever tip. *P*1, *P*2 and *P*3, starting or ending points of the scanning; trace, *P*1 and *P*2, along downward direction (towards digestive zone); retrace, *P*2 and *P*3, along upward direction (towards peristome). *L*/*W*, length/width of the scanning area; *d*, scanning width, equal to the diameter of cantilever tip $d_{T}$, 30 nm; *α*, angle between scanning trajectory and scanning area, $\tan\alpha =d_{T}/L$, so *α* is 0.43°; *β*, angle of the cantilever tip, 15°; $F_{L}$, load force; WC, wax crystals; LC, lunate cell.

Mature pitchers (figure 1*a*, length 130.8 ± 5.5 mm, *n* = 12, mean ± s.d. used throughout) of *Nepenthes alata* were selected to investigate the surface anisotropy of slippery zone. The *N. alata* plants were commercially obtained from a nursery. Four species of adult insects were chosen to study their climbing behaviours in the slippery zone. Ant *Camponotus japonicus* was acquired from a commercial supplier, the rest (fly *Musca domestica*, moth *Noctuidae insect* and ant *Monomorium pharaonis*) were captured. Their body weight and length are shown in table 1.

**Table 1.** Parameters of the experimental insects (*n* = 16).

insects	body weight (mg)	body length (mm)	claw tip radius (µm)
fly *Mu. domestica*	17.51 ± 2.31	7.64 ± 0.21	1.46 ± 0.17
moth *Noctuidae insect*	31.64 ± 3.20	13.82 ± 0.69	2.03 ± 0.12
ant *Cam. japonicus*	21.16 ± 0.98	12.29 ± 0.42	3.18 ± 0.43
ant *Mo. pharaonis*	0.46 ± 0.02	3.26 ± 0.14	1.51 ± 0.16

### 2.2. Climbing behaviours observation

Almost half of the pitcher was longitudinally removed, the rest still connecting to the *Nepenthes* plant was used for the climbing behaviour experiment. This design was much better than the previous designs [22,25,37], causing the climbing route to be observed and recorded clearly, and avoiding the structural shrink caused by water evaporation. As shown in figure 1*a*, the pitchers were fixed in their normal and inverted direction, respectively. The insects were put on the starting region, then climbed forward along the upward (towards peristome) and downward (towards digestive zone) directions. Their climbing behaviours were recorded by a digital camera (Nikon D90, Nikon Co.) to analyse the effect of direction on the displacement and velocity. The experiment was conducted under a temperature of 28°C and a relative humidity of 35%.

### 2.3. Friction force measurement

Among our selected insects, claws of the fly *Mu. domestica* are not their decisive attachment organs, claws of the moth *Noctuidae insect* are basically concealed by the conspicuous arolium, claws from ant *Cam. japonicus* and ant *Mo. pharaonis* are their rather important attachment organs; however, claws of the ant *Mo. pharaonis* are rather small to increase the operation difficulty of friction experiment. Claws of the ant *Cam. japonicus* present relatively larger structures, thereby being selected for the friction measurement. Friction force of insect (ant *Cam. japonicus*) claws in slippery zones was measured with a homemade friction measurement apparatus. The structure and operating procedure have been described in detail in previous literature [38–40]. Claws and slippery zones (15 × 15 mm) were fixed on silicon wafers (20 × 20 mm), then fixed to the horizontal and vertical cantilever (figure 1*b*), respectively. Further, the slippery zone was fixed in its normal and inverted direction, respectively. The horizontal cantilever first moved towards the vertical cantilever (*d*1 direction) to contact the inverted-/normal-fixed slippery zone and form a normal deformation, then moved downward (*d*2 direction, 0.05 mm s^−1^) to generate a frictional deformation. The deformations were detected by the sensors, and the normal force and friction force could be achieved via the calibration experiment. The measurement was conducted under a temperature of 28°C and a relative humidity of 35%.

### 2.4. Atomic force microscope scanning

Scholz *et al*. [41] firstly scanned the wax crystals with an AFM apparatus, presenting the value of load force which the wax crystals could withstand. In our AFM scanning, the slippery zone from *N. alata* plant was selected as the experimental sample, the scanning direction (downward or upward) and the values of load force (100–3000 nN) were the variable factors. The friction data provided by the AFM cantilever could provide original evidence to characterize the surface properties (anisotropy or isotropy) of wax crystals. For the scanning, an AFM apparatus (MFP-3D Classic, Oxford Instrument Co.) and a high reflection-coated triangular silicon nitride cantilever (Multi75E-G, Nanoworld, figure 1*c*) were used. The AFM scanning was performed to obtain (i) friction data of the cantilever tip on wax crystals, and (ii) damage degree of the wax crystals generated by the cantilever tip. A sample (2 cm^2^) was cut from the slippery zone and glued to a circular glass slide, then scanned in contact mode with a constant velocity of 2 µm s^−1^. When scanning on the wax crystals, friction data can be achieved. As shown in figure 1*c*, moving direction of the AFM cantilever in trace and retrace was along the downward (towards digestive zone) and upward direction (towards peristome), respectively. As the scanning process was frequently interrupted by the lunate cells having a height of approximately 10 µm [25,27], the scanning area was adjusted to 4 × 1 µm. In another aspect, to easily find the damage region of wax crystals, the scanning area was adjusted to 20 × 10 µm. The scanning was conducted at a temperature of 25°C and a relative humidity of 25%.

### 2.5. Scanning electron microscope observation

Samples (2 cm^2^) from the pristine and scanned slippery zones were dried with a method of critical point drying, and sputter-coated (Gatan 682 PECS, Gatan Inc.). To exhibit the original morphology, samples without the critical point drying and the sputter-coating were also prepared. Attachment organs were removed from the experimental insects and air-dried, then mounted to alloy holders and sputter-coated. These samples were observed with an SEM apparatus (Hitachi S-4800N, Hitachi Co.). Structural parameters were estimated by analysing the SEM images. The scanning was conducted at a temperature of 25°C and a relative humidity of 25%.

## 3. Results

### 3.1. Microstructures of slippery zone and attachment organ

**Figure 2.**
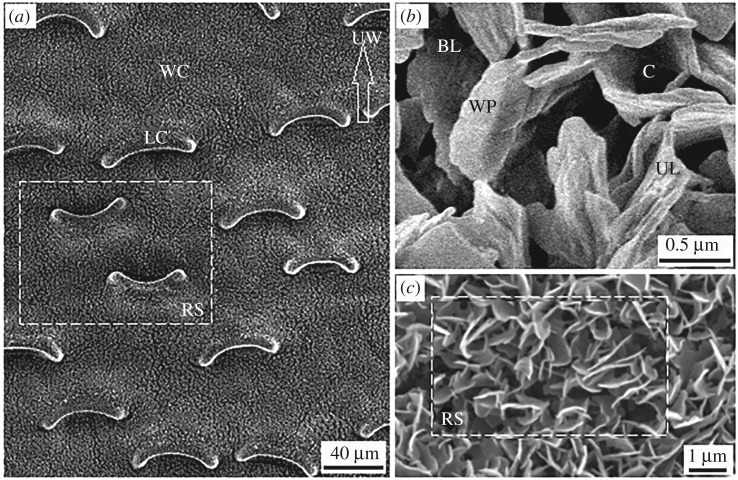
Micrographs of the slippery zone. (*a*) The unprocessed slippery zone, a white dotted rectangle region showed the morphology of the inverted slippery zone (RS). WC, wax crystals; LC, lunate cell; UW, upward. (*b*) Waxy platelets (WP), forming cavities (C) and an upper layer (UL), some regions showed a beneath layer (BL). (*c*) The morphology of the inverted wax crystals (RS). The wax platelets and cavities were simplified as wafers, and the radius of wax platelets $R_{\mathrm{WP}}$ = 173.4 ± 14.0 nm and cavities $R_{C}$ = 467.6 ± 36.7 nm, *n* = 20. The height of the wax crystals $H_{\mathrm{WP}}$= 237.1 ± 11.8 nm, *n* = 16. Space length and density of lunate cells were 94.3 ± 8.5 µm and 266.6 ± 19.7 mm^–2^, respectively, *n* = 20.

Similar to previous results [25,28,42,43], our micrographs of the slippery zone showed a layer of wax crystals, along with a large number of scattered lunate cells (figure 2*a*). The wax crystals are composed of discernible wax platelets with irregular profile, mostly overlapping each other to form undistinguishable cavities (figure 2*b*). These wax platelets and cavities constitute an upper layer, and an indistinctive beneath layer exists under the upper layer (figure 2*b*). The lunate cell corresponds to an enlarged guard cell with both ends bending downward, forming a crescent-formed profile (figure 2*a*). When inverting the slippery zone, the difference in topography of lunate cells was obviously exhibited (figure 2*a*), whereas the difference in wax crystals could hardly be detected (figure 2*c*). Moreover, wax crystals without sample treatment (critical point drying and sputter-coating) showed a rather similar topography in the two directions (figure 2*a*).

**Figure 3.**
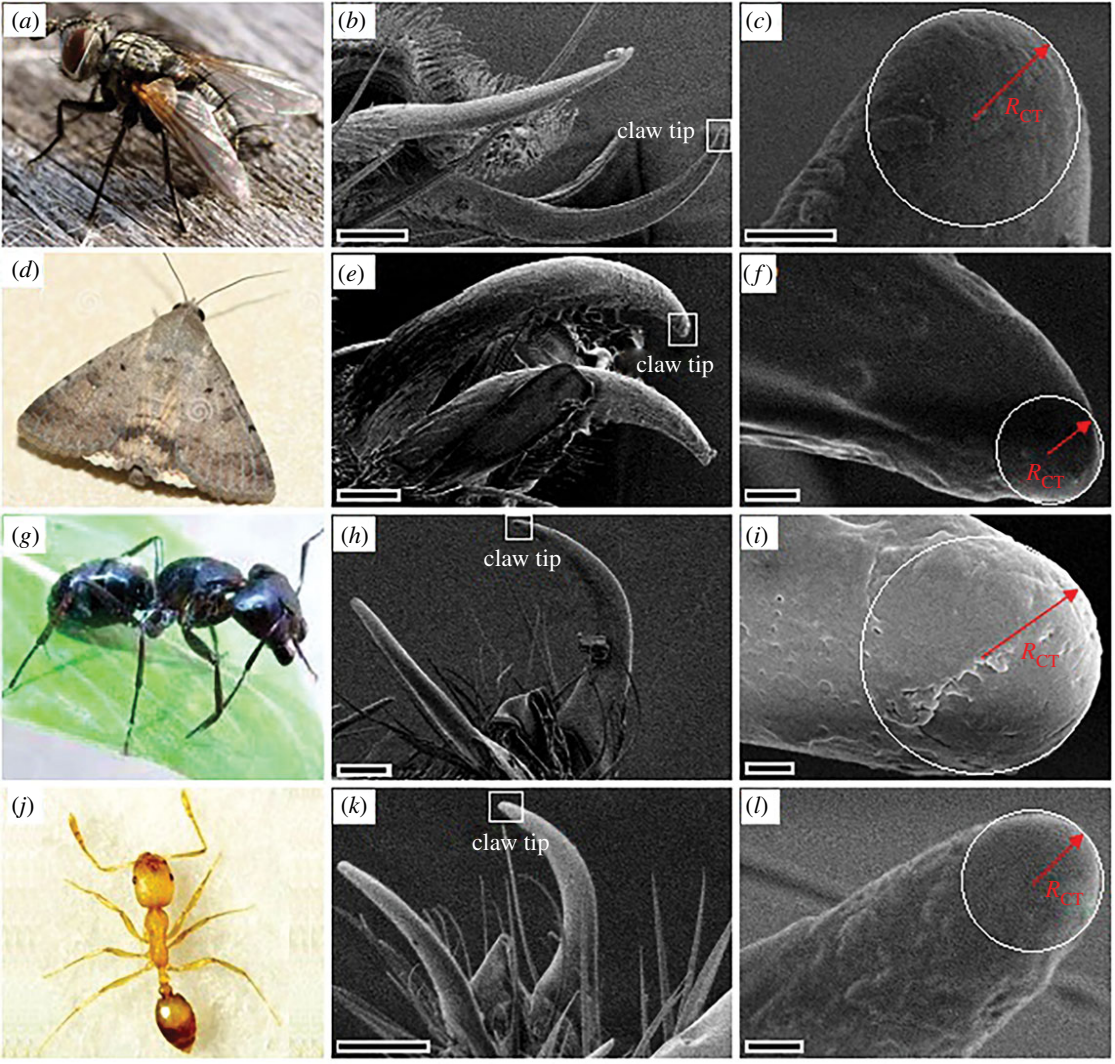
Attachment organs. (*a*,*d*,*g*,*j*) The selected insects. (*b*,*e*,*h*,*k*) General morphology of claws, black bars represent 25 µm. (*c*,*f*,*i*,*l*) Hemisphere-shaped claw tips, black bars represent 1 µm. $R_{\mathrm{CT}}$, claw tip radius. (*a*–*c*) Fly *Mu. domestica*. (*d*–*f*) Moth *Noctuidae insect*. (*g*–*i*) Ant *Cam. japonicus*. (*j*–*l*) Ant *Mo. pharaonis*.

These ventrally curved claws showed similar hemisphere-shaped tips (figure 3). Although insects showed a 68.8 times difference in their body weights, the difference in their claw tip radii was only within 2.2 times (table 1). When the substrate irregularity radius is larger than the claw tip radius, claws can generate mechanical interlock, contributing to the locomotion and attachment of insects [44–46]. Thus, these micron-scaled claw tips are very beneficial to generating an effective attachment to various substrates.

### 3.2. Climbing behaviours of insects

Previous studies have shown the statistical data of insects climbing in the slippery zone. All flies *Cal. vomitoria* and ants *Cam. japonicus* failed to climb across the upward slippery zone despite numerous attempts, but could climb across the inverted slippery zone easily [22,25]. About 6% of experimental ants *Mo. pharaonis* could climb up the upward slippery zone, but 58% could climb across the downward one [37]. In the following, we provide the procedure information (displacement, velocity) to further describe the anisotropy.

When on inverted-fixed slippery zone (figure 1*a*), insects could climb up (towards digestive zone) without effort and most of them gradually reached the digestive zone. However, none of the insects could climb up the normal-fixed slippery zone (figure 1*a*, towards peristome), despite massive attempts. Most of the insects continually scrambled on the borderline between the slippery zone and the digestive zone, but less than half of their bodies could move across the borderline. These opposite phenomena behaviours suggest that the normal-fixed slippery zone can invalidate the climbing behaviour of insects, whereas the inverted-fixed slippery zone makes insects present an excellent climbing capability.

**Figure 4.**
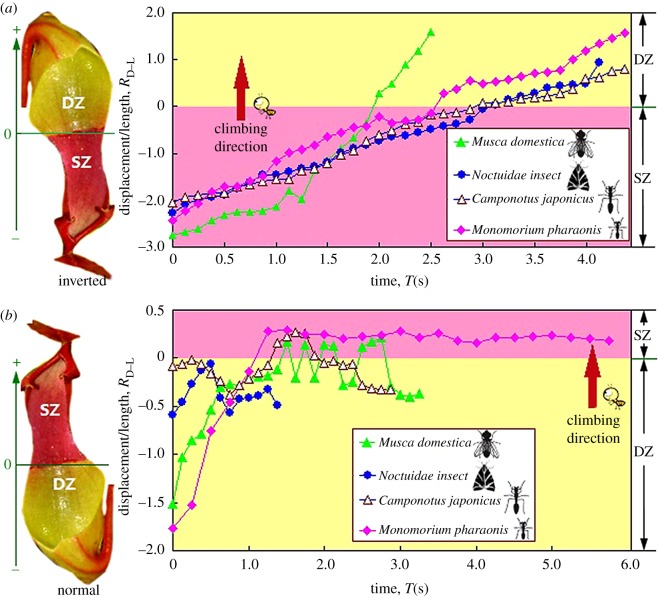
The ratio of displacement to body length. Zero points of longitudinal coordinates equal to the borderline between the slippery zone (SZ) and the digestive zone (DZ). (*a*) Inverted-fixed pitcher. (*b*) Normal-fixed pitcher.

By analysing the climbing behaviours, we obtained the displacements, and further calculated the ratio of displacement to insect length, named $R_{D-L}$. On inverted-fixed pitchers (figure 1*a*), insects generated high $R_{D-L}$ values (figure 4*a*), and gradually climbed across the slippery zone without obvious difficulty. On normal-fixed pitchers (figure 1*a*), insects rapidly climbed across the digestive zone to reach the borderline of slippery zone, producing high $R_{D-L}$ values (figure 4*b*). Then, they scrambled on the borderline to attempt to climb in the slippery zone, but less than half of their bodies achieved, so the $R_{D-L}$ values were rather small (lower than 0.3, figure 4*b*). The $R_{D-L}$ values continuously increased when insects climbed on the inverted-fixed pitchers, indicating they could always climb forward. Whereas on the normal-fixed pitchers, insects frequently slid backward, so the $R_{D-L}$ values showed an up-and-down trend.

**Figure 5.**
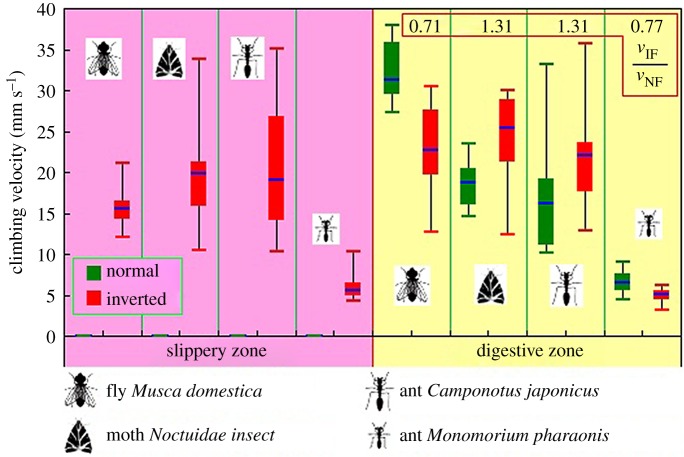
Climbing velocity, *n* = 18. IF, inverted-fixed pitcher, climbing from the slippery zone to the digestive zone; NF, normal-fixed pitcher, climbing from the digestive zone to the slippery zone.

Further, the climbing velocities were obtained (figure 5). As insects could not completely climb in the normal-fixed slippery zone, the velocities were zero. In the inverted-fixed slippery zone, insects provided high velocities (6.16–20.47 mm s^−1^). Without doubt, it is the anisotropy of the slippery zone to cause the conspicuous difference. In the digestive zone, the direction produced little effect, as the velocities in inverted-fixed pitchers were 0.71–1.31 times those in normal-fixed pitchers. The digestive zone consists of multicellular glands with downward hoods, resulting in a remarkable anisotropy. However, the anisotropy slightly contributes to the insect attachment, because material stiffness of the digestive zone permits the claws to cling [33].

According to these findings, it can be deduced that the direction of slippery zone dramatically affects the climbing behaviours of insects, in terms of climbing displacement and velocity. As surface structures of the slippery zone consist of lunate cells and wax crystals, both of the two structures should make contributions to the anisotropy.

### 3.3. Friction force of insect claws

**Figure 6.**
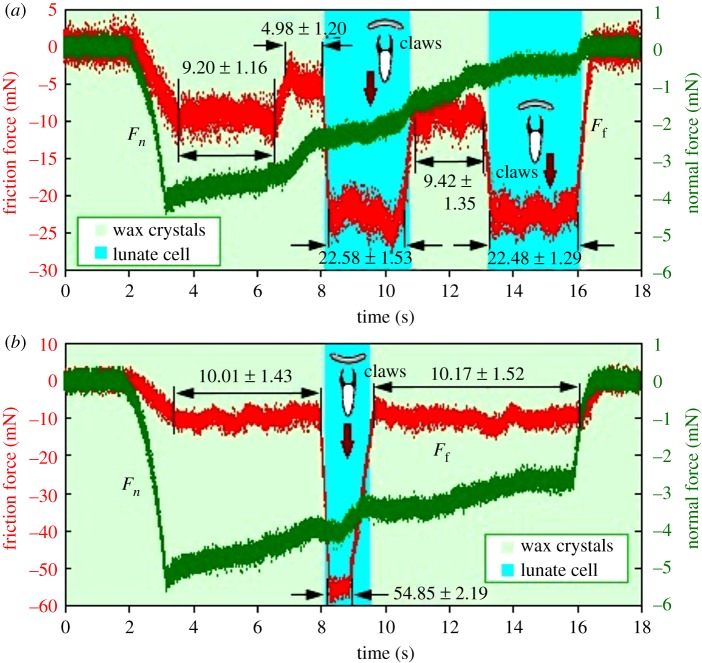
Friction force of insect (ant *Cam. japonicus*) claws in the slippery zone (normal force 4.5 mN). (*a*) Normal-fixed slippery zone. (*b*) Inverted-fixed slippery zone.

To further characterize the surface anisotropy, friction force of insect (ant *Cam. japonicus*) claws in the slippery zone was measured. Under a normal force, the claws contacted tightly with the slippery zone, and generated the friction force when being pulled downward. Lunate cells from both inverted- and normal-fixed slippery zones increased the friction force obviously, but the inverted-fixed ones presented a more significant degree. In the normal-fixed slippery zone, friction force of the lunate cells was 2.39–2.45 times that of the wax crystals, whereas in the inverted-fixed one, this ratio showed a much higher value (5.39–5.48, figure 6 and table 2). Similar results were obtained when a normal force of 1.5 mN was applied, presenting 1.89–2.26 and 4.44–4.70 times (electronic supplementary material, figure S2 and table S1), respectively. Further, when the claws were sliding in inverted-fixed lunate cells, the friction force was 2.4 times that in normal-fixed ones (figure 6 and table 2), and a similar result (2.1 times, electronic supplementary material, figure S2 and table S1) was presented when a normal force of 1.5 mN was applied. The results indicate that the lunate cells present a significant anisotropy, making insect claws generate much higher friction force when scrambling in the inverted-fixed slippery zone.

**Table 2.** Ratio of the friction force (normal force 4.5 mN). Note: $F_{f-\mathrm{NF}}$,$F_{f-\mathrm{IF}}$, friction force in the normal- and inverted-fixed slippery zone; $F_{f-\mathrm{LC}}$,$F_{f-\mathrm{WC}}$, friction force on lunate cells and wax crystals; $F_{N}$, normal force.

	lunate cells region			wax crystals region			normal-fixed	inverted-fixed
types	$F_{f-\mathrm{NF}}/F_{N}$	$F_{f-\mathrm{IF}}/F_{N}$	$F_{f-\mathrm{IF}}/F_{f-\mathrm{NF}}$	$F_{f-\mathrm{NF}}/F_{N}$	$F_{f-\mathrm{IF}}/F_{N}$	$F_{f-\mathrm{IF}}/F_{f-\mathrm{NF}}$	$F_{f-\mathrm{LC}}/F_{f-\mathrm{WC}}$	$F_{f-\mathrm{LC}}/F_{f-\mathrm{WC}}$
values	5.00–5.02	12.33	2.40	2.04–2.09	2.22–2.26	1.06–1.11	2.39–2.45	5.39–5.48

When the claws slid on the inverted- and normal-fixed wax crystals, an unobvious difference in the friction forces was shown, namely 1.06–1.11 times (normal force 4.5 mN, table 2) and 1.03–1.05 times (normal force 1.5 mN, electronic supplementary material, table S1), respectively. The result indicates the wax crystals exhibit slight contribution to the surface anisotropy of slippery zone. Moreover, the claws provided a much higher ratio of friction force to normal force with the normal force of 1.5 mN than that with the normal force of 4.5 mN (table 2; electronic supplementary material, table S1). As the normal force of 1.5 mN is equal to a 7.2 times of the insect body weight (21.2 mg, table 1), it is probably close to the saturated value which makes the claws generate a maximal friction force. Therefore, the friction force cannot continuously increase with a redoubled tendency when the normal force is increased to 4.5 mN.

### 3.4. Friction test with an atomic force microscope cantilever

**Figure 7.**
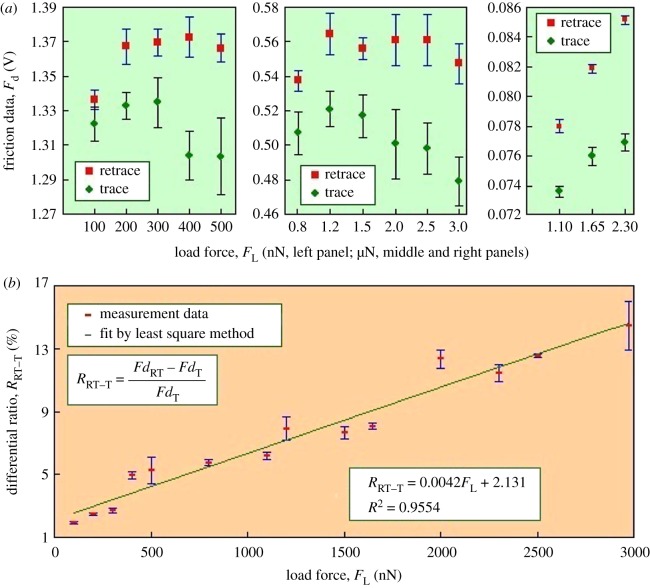
The difference in friction data provided by a cantilever tip scanned on slippery zones along the downward and upward directions. (*a*) Statistical values of the friction data under various load forces, statistical values, *n* = 16 384 (left and right) and 32 768 (middle). (*b*) Differential ratio values of the friction data, *n* = 4.

After the AFM scanning, the trace and retrace images were obtained and analysed with software (Igor Pro V6.36, Oxford Instrument Co.), exporting the friction data of cantilever tip on wax crystals along downward and upward directions. The friction data provide a possibility to judge the surface properties (anisotropy or isotropy) of wax crystals. When given a load force, the friction data were different (electronic supplementary material, figure S3). These differences were rather small when the load forces were 100, 200 and 300 nN, but became obvious when increasing the load force from 400 to 3000 nN. Further, under all the applied load forces, the friction data along the upward direction (retrace, towards peristome, figure 1*c*) were invariably higher than those along the downward direction (trace, towards digestive zone, figure 1*c*). It is noteworthy that the friction data frequently decrease with the increase in the load force (figure 7*a*). When the applied load force exceeds a specific value, the cantilever tip can destroy the wax crystals and the fractured wax crystals contaminate the cantilever tip, severing as a lubricant to cause the decrease in the friction data. As demonstrated in previous literature [23,24], the wax crystals can contaminate insect attachment organs and decrease the attachment force of insects.

With the increase in the load force, the difference in the two types of friction data became more observable. To quantitatively describe the difference, values of differential ratio $R_{\mathrm{RT}-T}$ were calculated (figure 7*b*). The $R_{\mathrm{RT}-T}$ values grew gradually with the increase in load force, showing a linear trend. Further, when the load forces of 100–300 nN were applied, the $R_{\mathrm{RT}-T}$ values were rather small (1.92–2.72%), and became more noticeable (4.96–14.63%) when increasing the load force (400–3000 nN). Accordingly, when being scanned along the upward and downward directions, the wax crystals make the cantilever tip generate different friction data, but these differences are not conspicuous, even rather unobvious.

### 3.5. Damage degree of scanned wax crystals

**Figure 8.**
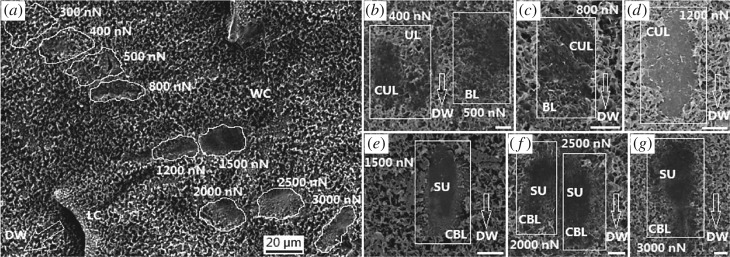
Damage degree of the wax crystals scanned by the cantilever tip. (*a*) General micrograph. (*b*–*g*) Partial micrographs showing the definite morphology of damaged wax crystals. UL/BL, upper/beneath layer; CUL/CBL, collapsed upper/beneath layer; SU, substrate of the slippery zone; DW, downward (towards peristome). The white bars in *b*–*g* represent 5 µm.

Morphology of the scanned wax crystals was observed, investigating the damage degree caused by the cantilever tip under various load forces. When the cantilever was applied different load forces, the damage degree was significant different (figure 8). No detectable damage was presented when the load forces of 100 and 200 nN were applied, and a negligible damage was observed when the load force of 300 nN was applied (figure 8*a*). A previous study presented a similar result, as the load force up to 250 nN could not destroy the wax crystals, but higher load force was not tested [41]. When increasing the load force (400–1200 nN), wax crystals showed observable damage, and the degree became much larger. On the scanned regions (figure 8*b*–*d*), the upper layer of wax crystals fractured and collapsed, generating a collapsed upper layer. Some destroyed regions showed the beneath layer. These results indicate the cantilever tip can destroy the upper layer under the load forces of 400–1200 nN. When continuously increasing the load force (1500–3000 nN), the beneath layer fractured, showing a collapsed beneath layer. On some regions, the two layers were completely destroyed, exposing the substrate of slippery zone (figure 8*e–g*). That is, the cantilever tip can destroy the entire structures of wax crystals when the load force is higher than 1500 nN.

## 4. Discussion

### 4.1. Contribution of lunate cells to anisotropy

Without doubt, the lunate cells make the slippery zone present surface anisotropy depending on their anisotropic configuration. This anisotropy makes the slippery zone present rather different insect attachment behaviour and wettability along the downward (towards digestive zone) and upward (towards peristome) orientation [25,36,37]. A mechanical analysis showed a theoretical explanation to the conspicuous difference in attachment behaviour, as the lunate cells depended on their small slope angle (about 23°) to preclude the insect claws from generating mechanical interlock, whereas the inverted ones relied on their large slope angle (about 76°) to enhance the mechanical interlock [25]. In our present study, detailed procedure information, including displacement, velocity and real-time friction force, were provided to further characterize the anisotropy caused by lunate cells. Insects could not climb ahead (towards peristome) in the normal-fixed slippery zone, but showed effortless climbing behaviour (towards digestive zone) in the inverted-fixed slippery zone and generated high displacements (figure 4) and velocities (6.16–20.47 mm s^−1^, figure 5). When applying the normal forces of 4.5 and 1.5 mN, friction force of insect claws sliding in inverted-fixed lunate cells (figure 1*b*) was 2.4 and 2.1 times that in normal-fixed ones (figure 1*b*), respectively. These innovative results showed much more convincing evidence to characterize the anisotropy of slippery zone.

Insect claws can generate mechanical interlock when the diameter of claw tip is smaller than that of the substrate irregularity [44]. The height of the lunate cells is about 20 µm, which is observably higher than the claw tip radius (1.46–3.18 µm, table 1). Further, the activity range of a single claw is normally higher than 1 mm, so the activity area exceeds 3.14 mm^2^. The high distribution density (247–286 mm^−2^) makes lunate cells provide numerous choices for insect claws to generate the mechanical interlock. According to this analysis, the lunate cells should guarantee the generation of mechanical interlock, making insects present effective climbing behaviours. In fact, the effortless and invalid climbing behaviours were shown when insects climbed in the inverted- and normal-fixed slippery zone, respectively. In the two directions, morphology of the lunate cells changed significantly (figure 2*a*), and the wax crystals showed no detectable difference (figure 2*c*). These phenomena suggest the lunate cells execute a rather important function in the surface anisotropy of slippery zone. However, based on these results, the contribution of wax crystals cannot be completely excluded.

### 4.2. Contribution of wax crystals to anisotropy

**Figure 9.**
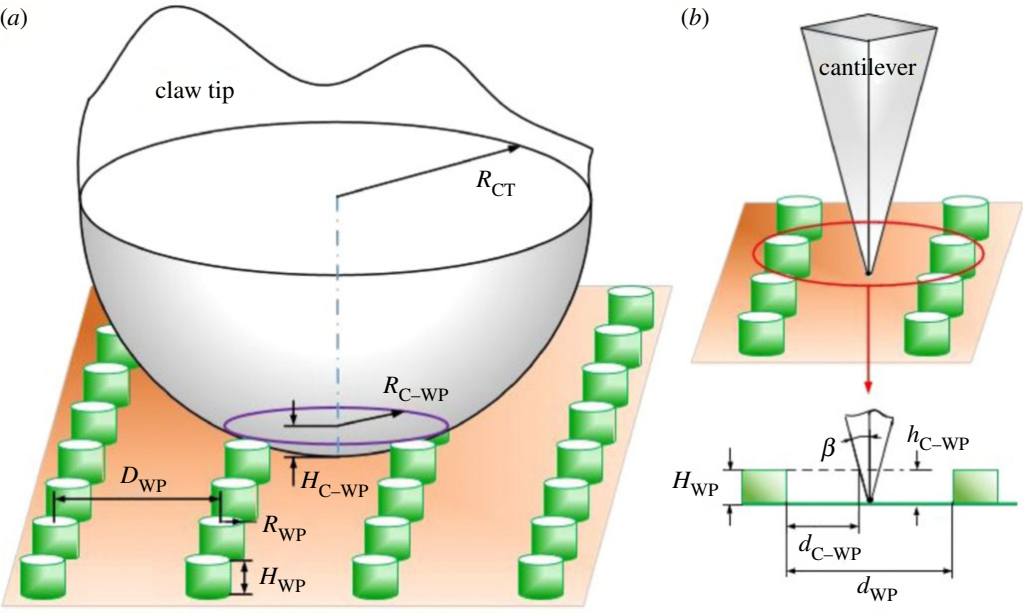
Schematics show the interactions. (*a*) Interaction with the claw tip. (*b*) Interaction with the cantilever tip. $R_{\mathrm{CT}}$, claw tip radius; $R_{C-\mathrm{WP}}$, radius of contact circle between the claw tip and the wax crystals; $H_{C-\mathrm{WP}}$, depth of the claw tip inserting wax crystals; $R_{\mathrm{WP}}$/$H_{\mathrm{WP}}$, wax crystals radius/height; $D_{\mathrm{WP}}$, distance among the wax crystals; β, cantilever tip angle, 15°; $h_{C-\mathrm{WP}}$, depth of the cantilever tip inserting wax crystals; $d_{C-\mathrm{WP}}$, distance between the cantilever boundary and the wax crystals; $d_{\mathrm{WP}}$, distance among the boundaries of wax crystals.

When insect claws slid on the inverted- and normal-fixed wax crystals, different friction forces were generated, but the difference was rather unobvious (figure 6; electronic supplementary material, figure S2). Different friction data were generated when the cantilever tip scanned on wax crystals along downward (trace, towards digestive zone) and upward (retrace, towards peristome) directions, and the differences became more observable when increasing the load force (figure 7). Interactions between the insect claws and the wax crystals, also between the cantilever tip and the wax crystals, could present visual evidence to describe the contribution of wax crystals to the anisotropy, so models were proposed to describe the interactions. In the models (figure 9), the wax crystals and their generated cavities were considered as an array of convex cylinders with an equal interval distance, and the structural parameters are shown in figure 2 and table 1.

According to the model (figure 9*a*), the distance among the boundaries of adjacent convex cylinders equals twice the cavity radius $R_{C}$, allowing the claws to insert the wax crystals with a depth $h_{C-\mathrm{WP}}$ = 35–77 nm, which is much smaller than their tip radii (1.46–3.18 µm, table 1). That is, the space formed by the cavities cannot accommodate the relatively bigger claw tip to generate the mechanical interlock. Further, the wax crystals connect to their adjacent layer via thin stalks and present a fragile property [23], which hardly withstands the lateral force provided by insects with high body weight, such as fly *Cal. vomitoria* [22], beetle *Adalia bipunctata* [23] and ant *Cam. japonicus* [25], excluding the possibility to form the mechanical interlock. When applying the normal forces of 4.5 and 1.5 mN, wax crystals hardly withstood the tremendous force (21.6 and 7.2 times body weight), so fractured. Therefore, in fact, the friction force was not provided by the surface morphology of wax crystals. Consequently, the difference (1.06–1.11 times, table 2; 1.03–1.05 times, electronic supplementary material, table S1) in friction forces provided by insect claws on inverted- and normal-fixed wax crystals (figure 1*b*) cannot serve as a convincing evidence to declare the anisotropy of wax crystals.

When the depth of cantilever inserting wax crystals $h_{C-\mathrm{WP}}$ equals the height of wax platelets $H_{\mathrm{WP}}$ (figure 9*b*), the distance between cantilever boundary and wax platelet $d_{C-\mathrm{WP}}$ is 404 nm, so the cavities can completely accommodate the cantilever tip. However, when applying the load forces of 100–300 nN, the differences in friction data along downward (trace, towards digestive zone) and upward (retrace, towards peristome) directions were rather unobvious (1.92–2.72%, figure 7*b*). Under these load forces, no detectable damage was exhibited on the wax crystals (figure 8*a*), suggesting the cantilever tip only slid on the surface topography of wax crystals. Therefore, the surface morphology of wax crystals cannot make the cantilever tip present anisotropic friction data. Therefore, the wax crystals hardly provide a substantial contribution to the anisotropy of slippery zone depending on their surface morphology. When increasing the load forces from 400 to 1200 nN, the differential ratios increased from 4.96 to 7.91% (figure 7*b*), and the cantilever tip destroyed the upper layer of wax crystals (figure 8*b*–*d*). As the two layers connect with each other via thin stalks, the structural feature probably provides the differences in the two types of friction data. However, the differential ratio is still too weak to be considered as strong evidence to declare the anisotropy. That is, the wax crystals cannot depend on their structural feature to present observable anisotropy. When increasing the load force from 1500 to 3000 nN, the differential ratio became much higher (7.62–14.63%, figure 7*b*). Under these load forces, results of the SEM observation showed that the cantilever tip could destroy the entire wax crystals completely, exposing the substrate of slippery zone (figure 8*e*–*g*). It is most likely that the substrate of slippery zone contacts with the cantilever tip and causes the generation of the friction data. Accordingly, these significant differences in friction data cannot serve as reasonable evidence to declare the anisotropy of wax crystals, because they probably result from the substrate of the slippery zone.

In addition, the AFM scanning presents essential evidence to analyse the climbing behaviours of insects. The body weight of ant *Mo. pharaonis* is 0.46 mg, meaning that only 0.38 µN is shared by each claw to act as the load force. This load force cannot cause the claw to destroy wax crystals observably. Further, the claw tip radius (1.51 µm, table 1) significantly exceeds the cavity radius (467.6 nm) of wax crystals, preventing the generation of mechanical interlock. Previous studies showed that the ant *Lasius niger* generated invalid climbing behaviour on the normal-fixed slippery zone, and no fractured wax crystals were observed on their attachment organs [37,41]. The above analysis probably provides a theoretical explanation to these phenomena. Body weight of the rest of the insects ranges from 17.5 to 31.6 mg (table 1), so each claw shares 14.3–25.8 µN to act as the load force. These forces are 9.5–17.2 times the load force of 1500 nN (figure 8*e*), indicating the claw can destroy the wax crystals completely. This analysis potentially shows the critical body weight of insects which destroys the wax crystals and causes contamination to attachment organs, as described in previous literature [22,23,25,28,47].

## 5. Conclusion

In this study, we showed macro–micro–nano scaled findings to analyse the contributions of lunate cells and wax crystals to the surface anisotropy of *Nepenthes* slippery zone. The inverted- and normal-fixed slippery zones made insects generate rather different climbing behaviours and friction forces, indicating the slippery zone possessed a conspicuous anisotropy. Analyses based on AFM scanning, microstructure observation and theoretical models suggested neither surface morphology nor structural characteristic could make the wax crystals show an observable anisotropic property. However, the lunate cells provided a remarkable contribution to the surface anisotropy depending on their anisotropic configuration. Our study has revealed the anisotropic mechanism of *Nepenthes* slippery zone, and provided theories to develop bioinspired surface with anisotropic properties.

## Supplementary Material

Supplementary Figures and Tables

## Supplementary Material

Data for the ratio of displacement to body length

## Supplementary Material

Data of the climbing velocity

## Supplementary Material

Data for the riction force of insect claws

## Supplementary Material

Data of the difference in friction data

## Supplementary Material

Data of Figure7 (AFM Scanning) 100-500nN

## Supplementary Material

Data of Figure7 (AFM Scanning) 800-3000nN

## Supplementary Material

Data of Figure7 (AFM Scanning) 1100-2300nN

## Data accessibility

All original data supporting this article have been uploaded as part of the electronic supplementary material.
