# Supplementary material for: Contributions of lunate cells and wax crystals to the surface anisotropy of Nepenthes slippery zone
Source: R Soc Open Sci. 2018 Sep 5;5(9):180766. doi: 10.1098/rsos.180766 (PMC6170553; doi:10.1098/rsos.180766)
Supplement: Supplementary Figures and Tables [file rsos180766supp1.doc]

**Supplementary Information for**

**Contributions of lunate cells and wax crystals to the surface anisotropy of *Nepenthes* slippery zone**

Lixin Wang^1^, Dashuai Tao^2^, Shiyun Dong^3^, Shanshan Li^1^ and Yu Tian^2^

^1^School of Mechanical Engineering, Hebei University of Science and Technology, Shijiazhuang 050018, People’s Republic of China

^2^State Key Laboratory of Tribology, Tsinghua University, Beijing 100084, People’s Republic of China

^3^National Key Laboratory for Remanufacturing, Academy of Armord Forces Engineering, Beijing 100072, People’s Republic of China

Authors for correspondence, Lixin Wang e-mail: wanglx@hebust.edu.cn

Yu Tian e-mail: tianyu@mail.tsinghua.edu.cn

**ORCID:** LW, 0000-0002-4205-5638; YT, 0000-0001-7742-5611


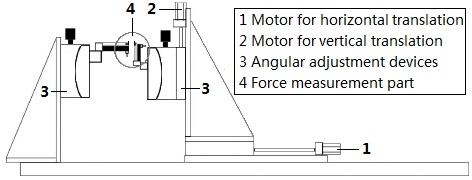


**Figure S1.** General structure of the friction measurement apparatus.


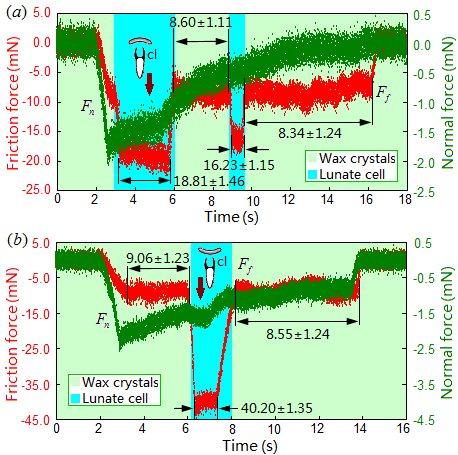


**Figure S2.** Friction force of the insect (ant *Camponotus japonicus*) claws in normal- and inverted-fixed slippery zones. The normal force applied to the insect claws is 1.5 mN. (*a*) In the normal-fixed slippery zone. (*b*) In the inverted-fixed slippery zone. cl, claws.

**
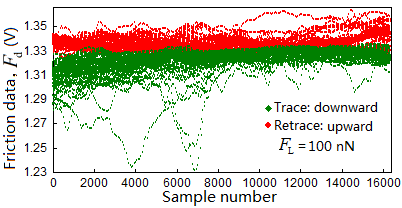
**

(*a*) Load force, $F_{\text{L}}$=100 nN

**
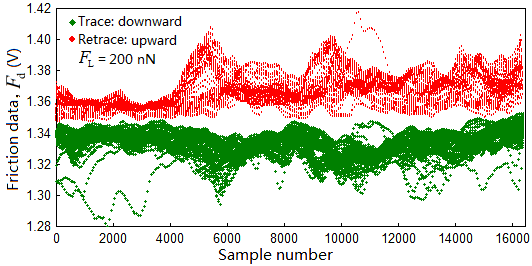
**

(*b*) Load force, $F_{\text{L}}$=200 nN

**
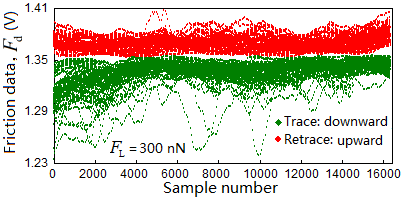
**

(*c*) Load force, $F_{\text{L}}$=300 nN


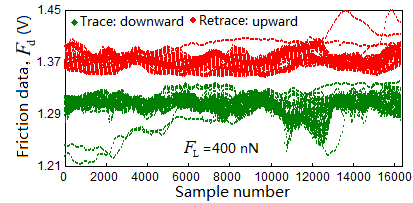


(*d*) Load force, $F_{\text{L}}$=400 nN

**
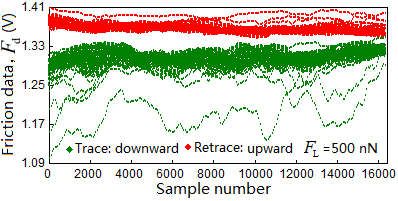
**

(*e*) Load force, $F_{\text{L}}$=500 nN

**
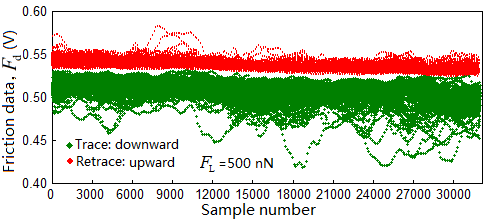
**

(*f*) Load force, $F_{\text{L}}$=800 nN

**
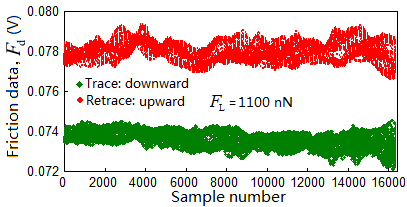
**

(*g*) Load force, $F_{\text{L}}$=1100 nN

**
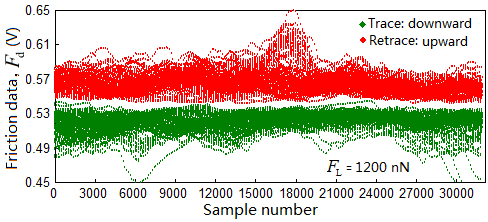
**

(*h*) Load force, $F_{\text{L}}$=1200 nN

**
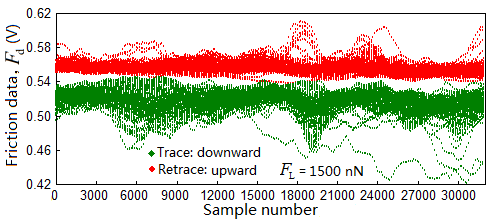
**

(*i*) Load force, $F_{\text{L}}$=1500 nN

**
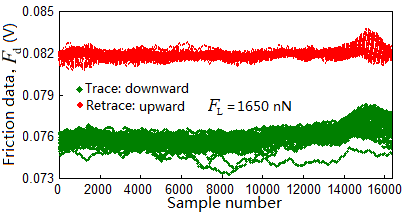
**

(*j*) Load force, $F_{\text{L}}$=1650 nN

**
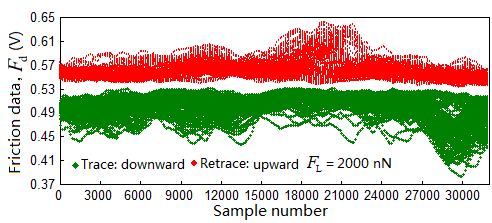
**

(*k*) Load force, $F_{\text{L}}$=2000 nN

**
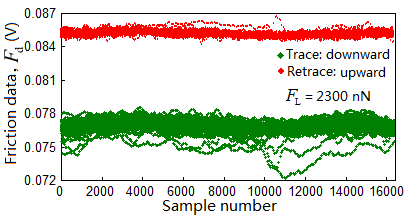
**

(*l*) Load force, $F_{\text{L}}$=2300 nN

**
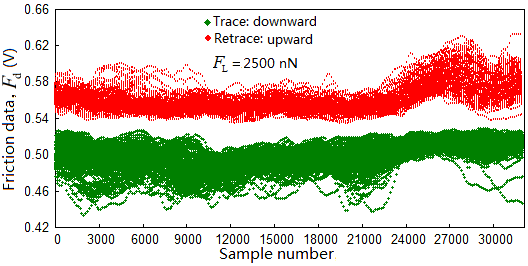
**

(*m*) Load force, $F_{\text{L}}$=2500 nN

**
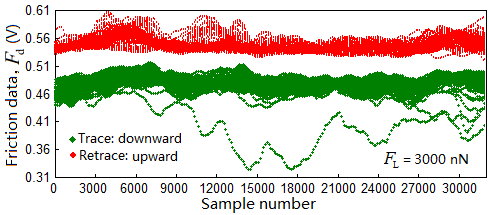
**

(*n*) Load force, $F_{\text{L}}$=3000 nN

**Figure S3.** Friction data of the cantilever tip on wax crystals along the downward (Trace, toward digestive zone) and upward (Retrace, toward peristome) directions. The load forces applied to the cantilever tip increased from 100 nN to 3000 nN. These images showed the difference in the friction data obtained from the Trace and Retrace scanning. We scanned the wax crystals in different times, and the cantilever was readjusted for each time, so the longitudinal coordinates were different.

**Table S1.** Ratio of the friction force (normal force 1.5 mN)

| Types | Lunate cells region $F_{\text{f-NF}}/F_{\text{N}}$ $F_{\text{f-IF}}/F_{\text{N}}$ $F_{\text{f-IF}}/F_{\text{f-NF}}$ | | | Wax crystals region $F_{\text{f-NF}}/F_{\text{N}}$ $F_{\text{f-IF}}/F_{\text{N}}$ $F_{\text{f-IF}}/F_{\text{f-NF}}$ | | | Normal-fixed $F_{\text{f-LC}}/F_{\text{f-WC}}$ | Inverted-fixed $F_{\text{f-LC}}/F_{\text{f-WC}}$ |
| --- | --- | --- | --- | --- | --- | --- | --- | --- |
| Values | 10.82-12.54 | 26.8 | 2.10 | 5.56-5.73 | 5.70-6.04 | 1.03-1.05 | 1.89-2.26 | 4.44-4.70 |

**Note:** $F_{\text{f-NF}}$,$F_{\text{f-IF}}$: friction force in the normal- and inverted-fixed slippery zones; $F_{\text{f-LC}}$,$F_{\text{f-WC}}$: friction force on lunate cells and wax crystals;$F_{\text{N}}$: normal force.
